# Acute Effects of Various Movement Noise in Differential Learning of Rope Skipping on Brain and Heart Recovery Analyzed by Means of Multiscale Fuzzy Measure Entropy

**DOI:** 10.3389/fnbeh.2022.816334

**Published:** 2022-02-25

**Authors:** Alexander Thomas John, Anna Barthel, Johanna Wind, Nikolas Rizzi, Wolfgang Immanuel Schöllhorn

**Affiliations:** Department of Training and Movement Science, Institute of Sport Science, Johannes Gutenberg-University, Mainz, Germany

**Keywords:** EEG, ECG, entropy, interaction, motor learning, physical activity, differential learning, recovery

## Abstract

In search of more detailed explanations for body-mind interactions in physical activity, neural and physiological effects, especially regarding more strenuous sports activities, increasingly attract interest. Little is known about the underlying manifold (neuro-)physiological impacts induced by different motor learning approaches. The various influences on brain or cardiac function are usually studied separately and modeled linearly. Limitations of these models have recently led to a rapidly growing application of nonlinear models. This study aimed to investigate the acute effects of various sequences of rope skipping on irregularity of the electrocardiography (ECG) and electroencephalography (EEG) signals as well as their interaction and whether these depend on different levels of active movement noise, within the framework of differential learning theory. Thirty-two males were randomly and equally distributed to one of four rope skipping conditions with similar cardiovascular but varying coordinative demand. ECG and EEG were measured simultaneously at rest before and immediately after rope skipping for 25 mins. Signal irregularity of ECG and EEG was calculated via the multiscale fuzzy measure entropy (MSFME). Statistically significant ECG and EEG brain area specific changes in MSFME were found with different pace of occurrence depending on the level of active movement noise of the particular rope skipping condition. Interaction analysis of ECG and EEG MSFME specifically revealed an involvement of the frontal, central, and parietal lobe in the interplay with the heart. In addition, the number of interaction effects indicated an inverted U-shaped trend presenting the interaction level of ECG and EEG MSFME dependent on the level of active movement noise. In summary, conducting rope skipping with varying degrees of movement variation appears to affect the irregularity of cardiac and brain signals and their interaction during the recovery phase differently. These findings provide enough incentives to foster further constructive nonlinear research in exercise-recovery relationship and to reconsider the philosophy of classical endurance training.

## Introduction

Today’s fast-paced society is always in search of maximum performance and efficiency, particularly in human’s physical and cognitive capacities. Effects of physical activity (PA) especially on the cardiovascular system (Fagard, 1997; Lavie et al., 2015; Nystoriak and Bhatnagar, 2018) and cognition (Li et al., 2017; Voelcker-Rehage et al., 2017; Etnier and Chang, 2019; Hillman et al., 2019; Ludyga et al., 2020) are well known. More recently and next to the type, intensity, or duration of a PA (Tomporowski, 2003; Schneider et al., 2009a,b; Brümmer et al., 2011; Pesce and Audiffren, 2011; Chang et al., 2012; Cox et al., 2016), the psycho-physiological effects that are induced by motor learning approaches (MLA) receive growing interest, mostly regarding possible benefits for cognitive and (neuro-)physiological performance (Brady, 2008; Henz and Schöllhorn, 2016; Schöllhorn, 2016; Henz et al., 2018; John and Schöllhorn, 2018; Fuchs et al., 2020). Especially the investigation of the PA underlying internal body processes related to the (neuro-)physiological system is of emerging demand (Wymbs and Grafton, 2009; Lage et al., 2015; Wright et al., 2016; John and Schöllhorn, 2018; Pauwels et al., 2018; Tomporowski and Pendleton, 2018; Sai Srinivas et al., 2021). This study represents a follow-up study based on the investigation (John and Schöllhorn, 2018) of how a short bout of rope skipping conducted with different underlying MLAs influences the (neuro-)physiological system. In a within-subject design three different MLAs, i.e., repetitive learning (RL) and according to the differential learning approach (DL) (Schöllhorn, 2000) instructed variable as well as self-created variable learning, were compared considering electrical brain activity by means of the electroencephalography (EEG), heart rate variability (HRV), and rating of perceived exertion. Borg scale of perceived exertion (RPE) as well as rating of mental effort were significantly higher in both DL interventions as in RL, whereas pure physical effort did not reveal significant differences. Thus, higher cognitive workload in both DL approaches was assumed. Immediately post exercise, slightly greater changes in HRV of DL suggested a higher sympathetic activation portending higher cognitive demands compared to RL. Referring to EEG analysis, higher parietal and temporal alpha power was found in RL compared to both DL interventions. Consecutive recovery of up to 30 mins revealed also higher temporal, parietal, and occipital theta, alpha and beta power in contrast to DL. Recapitulating, next to indices of a reciprocal impact of RPE, heart and brain activity, it was concluded that already a single bout of short time rope skipping could promote brain states presumably beneficial for cognitive learning. But adding movement noise actively via coordinatively demanding tasks with the application of the DL approach was supposed to provoke an overload of the mental capacity. Here, active movement noise is understood as the intentional augmentation of movement variability in a rather constructive manner, as opposed to the nuisance traditionally interpreted as destructive. Based on prior findings, the question arose whether the instructional frequency of nearly one new task per second is responsible for overloading mental performance due to the added stress of time pressure during a cyclic exercise. This study takes up the issue by adapting the task instruction frequency and analyzing its induced changes. In regard to existing indicators of a reciprocal influence of (neuro-)physiological strain in rope skipping (John and Schöllhorn, 2018), a study conducting an exhaustive PA (John et al., 2020) found brain lobe specific correlations of EEG spectral power with peripheral physiology, i.e., HRV. Thus, further incentives of a possible interaction between the two biosystems, in terms of brain and heart activity were given. With respect to the communication between the (neuro-)physiological systems and its accepted four major ways (McCraty, 2015), i.e., the biochemical, biophysical, energetic and neurological communication, here an interaction between both biosignals is seen in a neurological way, i.e., processes of the nervous system. However, the explanatory power of an interaction analysis via correlation of different analysis parameters based even on different biosignals, as conducted in John et al. (2020), has to be handled with caution. Investigating an interaction between brain and heart activity proves problematic due to various signal content between the (neuro-)physiological systems (Sik et al., 2017). Furthermore, different data preprocessing, e.g., band-pass and artifact filtering, as well as calculation techniques, e.g., time- versus frequency domain, dependent on the respective brain or heart measure aggravates besides varying value ranges of these parameters an interference-free interaction analysis. Thus, the application of a common signal processing and analysis procedure between (neuro-)physiological systems would be appreciated. This could be achieved using nonlinear analysis.

The most popular approaches for analyzing nonlinear signal characteristics investigate signal irregularity based on the entropy theory (Shannon, 1948). Commonly, irregularity of the EEG is characterized as signal noise, whereas it may contain valuable information (Sik et al., 2017) and seems relevant for a healthy, efficient and flexible neural function (Kosciessa et al., 2020). Regarding ECG, it is well known that the heart activity (HRV) needs to contain some noise or irregularity for being healthy (Peng et al., 1995). A suggested method for the interaction analysis of the brain and heart activity represents the signal entropy with its vast different types (Borowska, 2015; Jamin and Humeau-Heurtier, 2020). Low entropy values indicate a more deterministic signal with a high level of regularity or similarity, in contrast high values emerge in a less predictive signal with less regularity (Lin et al., 2017). Related to PA, entropy analysis of the brain and heart has only been investigated separately so far (Table 1). An interaction analysis between both biosignals using just entropy measures was only found in pure resting conditions without any physical demand. Accordingly, relevant previous research of entropy analysis is presented for both biosystems separately with a concluding description of the interaction studies.

**TABLE 1:** Overview of entropy research with analyzed electroencephalography (EEG), electrocardiography (ECG), and their interaction.

Study	Entropy type	PA	Intervention	Analysis		
				EEG	ECG	Interaction
Javorka et al., 2002	SampEn	+	Step-test		+*	
Lewis and Short, 2007	SampEn	+	Cycling		+*	
Ramanand et al., 2004	SampEn	+	Physical exertion (n.s.) and Cognitive tests	+		
Hogan et al., 2015	SampEn	+	Cycling and Cognitive tests	+		
Chuckravanen et al., 2015	InformationEn	+	Cycling and Cognitive tests	+		
Lin et al., 2017	FuzzyEn	+	Cycling	+		+**
Shi et al., 2017	ApEn, SampEn, FuzzyEn, PermEn, CE, and DistEn	+	Walking		+*	
Solis-Montufar et al., 2020	ApEn, SampEn, FuzzyEn	+	Walking		+*	
Lin et al., 2014	MultiscaleEn	–	Rest	+	+*	+
Li et al., 2016	ApEn	–	Cognitive tests	+		
Gao et al., 2016	WaveletEn	–	MBSR	+	+*	+
Sik et al., 2017	WaveletEn	–	MBSR	+	+*	+
Bhavsar et al., 2018	SampEn	–	Rest	+	+*	+

Chronologically ordered entropy research with analyzed EEG, ECG, or their interaction. PA, physical activity; + analyzed parameters. Entropy measures: ApEn, approximate entropy; SampEn, sample entropy; FuzzyEn, fuzzy entropy; PermEn, permutation entropy; CE, conditional entropy; DistEn, distribution entropy; InformationEn, information entropy; WaveletEn, wavelet entropy; MultiscaleEn, multiscale entropy; MBSR, mindfulness-based stress reduction; n.s., not specified. *Only HR or HRV entropy calculation was analyzed. **EEG ECG interaction with at least one non-entropy measure.

A field of extensive application was the analysis of the HRV-entropy. A walking study (Shi et al., 2017) compared HRV-entropy measures of a 5-min walking exercise on a treadmill at a regular speed with a resting seated position. Dependent on the entropy measure, differing effects were found with a comparable significant decrease of sample entropy as well as fuzzy entropy during walking. Related to a longer walking duration of 30 mins, HRV-entropy measures of sedentary and physical active subjects were compared to a prior resting condition (Solis-Montufar et al., 2020). For sedentary subjects, entropy values were high at rest and decreased with moderate physical activity. In contrast, physically active subjects revealed increased or related to rest stable entropies. Furthermore, longer physical demand seemed to slightly decrease entropies with emerging fatigue. According to more intense physical activity, a HRV study conducted an 8-min step-test of 70% of individual maximal power output with prior and posterior resting phases (Javorka et al., 2002). Sample entropy was marginally reduced after the exercise and reached pre-rest values after 25–30 mins of recovery. A graded exercise study on a bicycle ergometer (Lewis and Short, 2007) also measured HRV sample entropy before, during and after the cycling intervention. Sample entropy increased at the beginning of the physical workload, i.e., at light-to-moderate intensity, decreased with increments in exercise intensity and produced after a short gain a further decline in ongoing recovery.

Considering the entropy analysis of EEG, a cycling study (Lin et al., 2015) compared the effects of moderate and severe fatigue on EEG power spectrum and sample entropy. Severe fatigue appeared with incrementing EEG power in all frequency bands with continuing exercise. Sample entropy of electrode C3 attenuated gradually from resting to the last exercise session during moderate fatigue, and enhanced in severe fatigue during the late exercising stage (Lin et al., 2015). Another study (Ramanand et al., 2004) supports the use of entropy measures in EEG as a robust quantifier of complexity, applicable to detect variations induced by mental tasks or PA and the related fatigue. To investigate possible different effects of cardiorespiratory fitness and acute PA on executive functioning and EEG entropy, a study (Hogan et al., 2015) compared executive function performance and EEG sample entropy of higher and lower fit subjects after a bout of acute exercise or rest. No significant changes of acute exercise on EEG entropy were determined. EEG entropy of the left frontal area was significantly lower for higher fit participants compared to lower fit ones. The authors hypothesized that less fit subjects required greater effort eventually relying on higher levels of information processing, i.e., sample entropy (Hogan et al., 2015). Authors suggested that physical fitness could improve cognition by enabling higher functionality referring to lower levels of frontal EEG entropy. Another experiment found increasing approximate entropy (ApEn) of EEG with increments in task difficulty supposing ApEn applicable for evaluating of cognitive workload (Abhang et al., 2016). Regarding a possible relation between brain and heart activity, a study (Lin et al., 2017) investigated the effect of increasing heart rate during a tiring physical workload on EEG brain activity by means of EEG spectral power and fuzzy entropy. With increasing heart rate, spectral power of all measured electrodes, i.e., C1, C2, P1, and P2, similarly raised as fuzzy entropy portending a direct relation. But fuzzy entropy revealed superior specificity in determining frequency bands related to varying heart rate intensity and in addition provides a better computational efficiency.

Considering the analysis of the interaction between brain and heart activity, only studies correlating brain and heart entropies with no relation to PA were found. A study (Sik et al., 2017) investigated a relation of the (neuro-)physiological systems comparing a mental exercise with a resting condition. A linear correlation between brain and heart rate wavelet entropy was found during the mental exercise in contrast to the resting condition, particularly in central brain regions. Both, brain and heart rate entropy decreased compared to an eye-closed resting state. Therefore, irregularity of brain and heart activity was lowered indicating a higher coordination during mental exercise between the two biosignals, especially in somatosensory brain regions. A similar reduction of EEG and heart rate entropy was determined during mindfulness-based stress reduction training, but particularly in the occipital and frontal lobes (Gao et al., 2016). Also in a pure resting condition without any intervention, a low positive correlation between EEG, in frontal as well as somatosensory brain regions, and heart RR interval complexity based on sample entropy was found (Bhavsar et al., 2018). In contrast, a multiscale entropy study (Lin et al., 2014) revealed at rest an inversely correlated effect between RR intervals and EEG time signal at frontal, central and temporal regions. These opposing results could be due to multiple time scales present in and between biosignals (Humeau-Heurtier, 2020; Platisa et al., 2020), such as the brain (Papo, 2013; Hari and Parkkonen, 2015) and heart signal (Costa et al., 2005; Porta et al., 2009). The measure of sample entropy includes the disadvantage of only taking one time scale, i.e., the raw scale, into account for entropy calculation. Conversely, the multiscale entropy analysis (Costa et al., 2005) includes an adaptation of the time scale of the underlying signal to accentuate time scale dependent signal characteristics in entropy. In consequence, a quantitative comparison of interaction effects of heart and brain entropy with differing underlying time scales becomes feasible. Via correlating multiscale entropy values of EEG and ECG data, a possible relation of brain and heart signal irregularity considering the time scale characteristic of each biosignal could be declared. Whether the correlating effects of previous studies are persistent in general and in context with PA, needs further research.

This study aimed on one side to investigate the combined neuronal and physiological effects of single bouts of cyclic exercise-sequences with increased coordinative demand, i.e., rope skipping, that are based on various MLAs. Another purpose was to examine in particular whether the MLA dependent frequency of additional movement noise has an impact on the heart brain interaction. EEG brain and ECG heart activity by means of the nonlinear analysis method of multiscale fuzzy measure entropy (MSFME) were compared directly prior and after the PA. The underlying MLAs were repetitive learning and three chaotic DL approaches with differing frequencies of task instruction. The fact that previous study results were only based on the examination of highly automatized movements without a still existing noteworthy coordinative demand, i.e., cycling or walking, aggravates deriving hypotheses for the investigation of a highly coordinative PA like rope skipping. Additionally, mostly only a selection of particular brain regions of interest was analyzed and no consistent entropy measures were used. Based on this alternative type of analysis, i.e., applying a time scale specific interaction analysis between two biosignals with different time characteristics based on entropy, hypotheses were defined in rather general terms.

According to the results of the previous studies (Lewis and Short, 2007; Lin et al., 2017), we hypothesized increased general EEG and decreased ECG entropy independent of motor learning condition at rest, immediately after exercise termination. During recovery, entropy of ECG may increase slowly toward pre-rest value (Javorka et al., 2002; Lewis and Short, 2007). Due to missing references on EEG entropy results after a physical load in recovery, we assumed a subsequent monotone reduction of EEG entropy toward pre-exercise level. Furthermore, according to the different motor learning conditions, we hypothesize higher general entropy levels in all DL conditions due to additional cognitive workload compared to RL (Abhang et al., 2016; John and Schöllhorn, 2018). Referring to the sparse state of research concerning an interaction analysis between the brain and heart signal by means of (multiscale) entropy, hypotheses could only be defined undirected and based on separate EEG or ECG entropy results. Because of the additional cognitive load in DL, differences in the correlation of brain and cardiac multiscale entropy, i.e., the relationship between brain area and time-scale dependent brain and cardiac entropy, are hypothesized to occur immediately after training and in subsequent recovery between MLAs.

## Materials and Methods

### Participants

A total of 32 healthy right-handed male volunteers between 18 and 40 (27.3 ± 4.7) years participated in this study. Participation criteria were selected regarding gender and age dependent effects on the brain (Wada et al., 1994; Wackermann and Matousek, 1998) and heart activity (Umetani et al., 1998; Kuo et al., 1999) as well as handedness related differences in brain activity (Serrien et al., 2006; Sun and Walsh, 2006). Volunteers classed themselves as neurologically and cardiologically healthy, without knowledge of any associated pre-existing medical conditions. No physical or cerebral activity influencing substances (Zschocke and Hansen, 2012), like coffee, alcohol or medication, have been consumed at least 12 h before the measurement. All subjects confirmed being able to perform common rope skipping. Subjects gave their written informed consent for study participation. For anonymity of personal data, subjects were coded with numbers. The study has been conducted in accordance with the Declaration of Helsinki (2013). Compliance with the ethical standards was approved of the local institutional ethics committee.

### Study Design and Procedure

The study was conducted at the Sports Institute of the Johannes Gutenberg University of Mainz. With a between-subject design, the effects of four different coordination related MLAs were investigated. Participants were randomly and equally distributed to one of the four conditions resulting in eight subjects per group (one subject of RL condition was excluded due to massive artifactual signal noise). *A priori* power analysis based on the lowest effect size of EEG results (Cohen’s *d* = 1.843) of a previous rope skipping study (John and Schöllhorn, 2018) resulted in a recommended sample size of at least seven participants per group. EEG brain activity and ECG were chosen as measurement parameters for aftereffects in the (neuro-) physiological system. The measurements were carried out under laboratory conditions. Changes in brightness, ambient noise and temperature were standardized or kept to a minimum.

**FIGURE 1:**
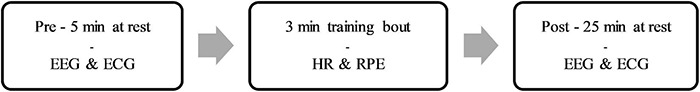
Test procedure.

The test procedure (Figure 1) started by measuring synchronized spontaneous EEG activity and ECG heart activity at rest for 5 mins just before the rope skipping training. The training bout was in accordance to a previous study (John and Schöllhorn, 2018) defined by 3 mins of rope skipping according to one of the underlying MLAs. During rope skipping, the heart rate (HR) was measured to check for any differences in the cardiovascular demand between MLAs. Prior to the training, subjects conducted a short rope skipping warmup of 15 s with a jumping frequency determined by an acoustic timer with 120 beats per minute. Perceived exertion (RPE) was rated directly after training termination. Immediately following, the recovery process was assessed during 60 mins at rest with synchronized EEG brain and ECG heart activity measurement. Measurements before and after rope skipping took place sitting on an immobile chair with eyes open facing to a white wall. Subjects were asked to sit comfortably, but also to minimize their head and eye movements. A mean duration of 112, SD 35 s was needed after the training before recovery measurement started. Due to partly loss of data during rest after the training bout, only the first 25 mins of the recovery process are used in this investigation.

### Apparatus

#### Motor Learning Approaches

The four different interventions were based on the interventions of the previous rope skipping study (John and Schöllhorn, 2018) and consisted of three DL variants with different levels of additional perturbations and RL. DL training generally uses movement variations to provoke a self-organized learning process by destabilizing the system via increased fluctuations, i.e., adding diffuse energy between two subsequent movement executions. This self-organized learning process is suggested to help finding an individually optimized solution for a certain physical activity problem that have to be adapted situationally. In contrast to RL, differences to the to-be-learned skill are not considered to be erroneous and detrimental to the learning process, but rather as essential fluctuations in living systems with a beneficial influence on learning. Hence, no repetition of an ideal, to-be-learned movement execution is recommended and in consequence no error correction has to be given (Schöllhorn, 2000).

All DL interventions relied on the chaotic DL model with verbal task instruction (John and Schöllhorn, 2018), but with a different frequency of task instructions. Based on the prior study, movement variations were applied by changes only in joint angles or their movements (Schöllhorn, 1999), e.g., feet crossed, head circling or knees flexed. All instructions were read aloud one after another depending on the predefined instruction frequency. The specific frequency of instructions was adequately practiced by the investigator prior to the start of the study to best possible ensure the compliance of the demanded frequency and to minimize variations in the frequency during the intervention. The condition names are chosen to present a general time continuous structure with the suffix of each DL condition representing the frequency of a new task instruction given. DL1 is defined by one task instruction nearly each second, which corresponds to a frequency of 1 Hz, resulting in maximal 180 different tasks. The other two DL interventions contained every ten (DL01), which corresponds to a frequency of 0.1 Hz, and every twenty seconds (DL005), which corresponds to a frequency of 0.05 Hz, one new task instruction resulting in 18 and nine different tasks, respectively. Each task should be performed until the next task instruction was given. Subjects were demanded to comply to a rope skipping frequency of 120 beats per minute, which corresponds to 2 Hz. RL was common, repetitive rope skipping with a frequency of 120 beats per minute and one ground contact per beat. Fitting to the structure of condition labeling, the RL condition was renamed to DL0 according to a variation frequency of 0 Hz, i.e., no given task instruction at all. The skipping rope was a steel rope including a bearing and individually adjusted to the anthropometric measures of the subject. In order to keep the psychological stress low in case of an interruption during rope skipping, the subjects were informed beforehand that they should simply resume the exercise as soon as possible. The total number of interruptions during the training intervention was documented.

#### Borg Scale of Perceived Exertion

The Borg rating of perceived exertion scale (RPE) (Borg, 1982) was applied to evaluate the exertion level between MLAs. Immediately after the training bout, subjects were asked to rate their individual RPE shortly. Subjects read an instruction of RPE 1 day and directly prior to the measurement beginning to ensure reliable exertion output. A RPE of 6 was defined as no effort at all and a RPE of 20 as maximum effort ever experienced.

#### Electroencephalography

Spontaneous resting EEG was assessed by means of the wired EEG-system Micromed SD LTM 32 BS (Venice, Italy) with a sampling rate of 1,024 Hz and recorded by the international 10–20 system using 19 electrodes, including Fp1, Fp2, F7, F3, Fz, F4, F8, T3, C3, Cz, C4, T4, T5, P3, Pz, P4, T6, O1, and O2. EEG was recorded before and after training sessions at rest. For all EEG measurements a homogeneous and low impedance (<10 kΩ) of the electrodes in all points was sought. The conduction of brain activity was unipolar with grounding on the nose. Furthermore, a two channel electrooculogram with electrodes at the medial upper and lateral orbital rim of the right eye was applied. To minimize artifacts such as EEG cable or cap movement caused by rope skipping, all loose EEG cables were fixed in the EEG cap or in the compressive net tubing, which was worn on the upper body of participants. Prior to each EEG measurement, the correct position of the EEG cap according to the 10–20 system was checked. Data was recorded by means of a commercially available software (SystemPlus Evolution - Micromed, Venice, Italy).

#### Electrocardiography

Electrocardiography was assessed by means of an additional bipolar channel of the EEG-system Micromed SD LTM 32 BS with a sampling rate of 1,024 Hz and recorded by the 2nd lead according to Einthoven. One electrode of the 2nd Einthoven limb lead was placed on the right side of the body, lateral, directly below the clavicle and the other on the left body side, lateral, directly below the costal arch to reduce artifacts during movement. ECG was recorded before and after rope skipping at rest by means of the software SystemPlus Evolution (Micromed, Venice, Italy). To reduce artifacts because of body or ECG cable movement, the upper body of participants was dressed up with a compressive net tubing. To assess the HR during rope skipping, the Polar watch RS800CX (Kempele, Finland) including a chest strap was used.

### Data Processing

Behavioral data was prepared for statistical analysis via Microsoft Excel 2019 (Microsoft, Redmond, WA, United States). Data of the recorded measurements of brain and heart activity were processed by means of MATLAB R2020b as well as MATLAB-based software EEGLAB 14_1_1b (MathWorks, Natick, MA, United States; Swartz Center of Computational Neuroscience, San Diego, CA, United States) (Delorme and Makeig, 2004).

#### Electroencephalography

A basic finite impulse response (FIR) filter was used to bandpass data (3–80 Hz). Power line noise (50 Hz) was filtered via spectrum interpolation (Leske and Dalal, 2019). Interference-prone channels were interpolated. Furthermore, an independent component analysis (ICA) (Makeig et al., 1996) was conducted. Recurring artifacts, such as eye closing, eye movement, and muscular artifacts were filtered by reducing interference-prone components. After visual inspection of the complete recordings, individually occurring abnormal interferences of the electric potential, like body movements or sweat artifacts, were eliminated out of the data length. After data cleaning, time signals were bandpass filtered via a minimum-order FIR filter to the total EEG spectrum of interest (4–70 Hz). Data of each electrode was down sampled to 256 Hz, split into nonoverlapping 10 s intervals and Z-transformed to efficiently calculate the entropy.

#### Electrocardiography

Electrocardiography data was bandpass filtered by a basic FIR filter (0.02–40 Hz). After visual inspection of the complete recordings, individual occurring abnormal interferences of the electric potential, like body movements or sweat artifacts, were eliminated out of the data length. Data was down sampled to 256 Hz, split into nonoverlapping 10 s intervals and Z-transformed.

Heart rate of the pre-exercise rest and during the rope skipping condition was averaged for each subject. To minimize individual differences in HR, averaged HR of the training bout was normalized by defining pre-rest HR as baseline (100%) and presenting HR of the training bout relative to the baseline (%Baseline) by dividing the training through the baseline HR. The difference between relative training HR and baseline was calculated to assess the change of relative HR due to the training intervention.

#### Multiscale Fuzzy Measure Entropy

**FIGURE 2:**
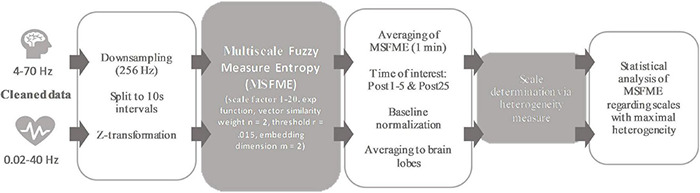
Data processing and analysis procedure.

**FIGURE 3:**
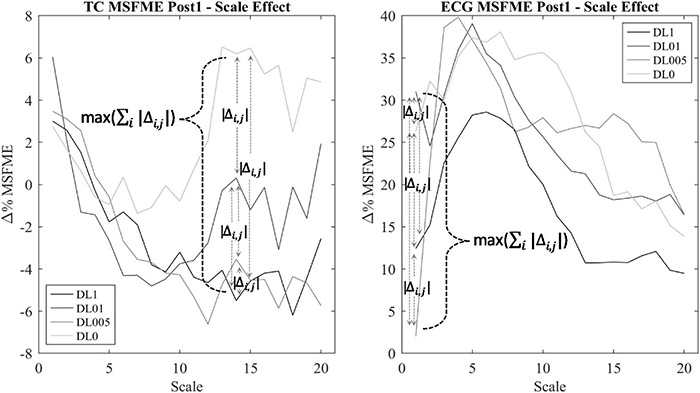
Scale determination via heterogeneity measure of TC multiscale fuzzy measure entropy (MSFME) Post1. Changes of baseline normalized MSFME values (Δ% MSFME) of all motor learning approaches (MLA)’s dependent on the scale interval (*i* = 1–20), index *j* for differentiation between pairwise condition choice of Δ% MSFME difference calculation (*j* = 1–6).

Regularity within EEG and ECG time signal was assessed via the MSFME (Figure 2) that is a combination of the fuzzy measure entropy (Chen et al., 2007; Liu et al., 2013) and the multiscale approach (Takahashi et al., 2009; Platisa et al., 2020). The fuzzy measure entropy was chosen due to the inclusion of local and global signal characteristics in the calculation of signal regularity and its better statistical stability compared to other entropy measures, like approximate and sample entropy (Chen et al., 2009; Liu et al., 2013). As fuzzy membership function, the exponential function with a vector similarity weight *n* of 2 and threshold *r* of 0.15 was used (Lin et al., 2017). The multiscale approach (Takahashi et al., 2009; Platisa et al., 2020) was applied to handle the different time characteristics of EEG and ECG time signals in the heart and brain interaction analysis. The scale factor τ was defined by the range of 1–20 (Takahashi et al., 2009; Platisa et al., 2020). MSFME was determined for every 10 s interval of the EEG and ECG time signal, containing 2,560 data values at scale 1 and 128 data points at scale 20. The embedding dimension m was set to 2, which is seen adequate for the analysis of at least 128 data points (Richman and Moorman, 2000; Chen et al., 2007). MSFME values of 10 s intervals were averaged to 1-min values. Baseline values were created relying on the last minute of pre-rest condition. Regarding the post-rest condition, 1-min average MSFME values were determined for the first 5 mins (Post1–5) to examine short-term recovery effects as well as for the last minute (Post25) to check for a remaining impact after total recovery time. To minimize individual differences, MSFME values of the post-rest condition were normalized by defining pre-rest MSFME as baseline (100%) and presenting post-rest MSFME relative to the baseline (%Baseline) by dividing the post-rest through the baseline MSFME. The difference between relative post-rest MSFME and baseline was calculated to assess the change of relative MSFME due to the training intervention during the recovery phase. EEG electrode specific MSFME values were averaged across their cerebral areas, i.e., FP1 and FP2 for the frontopolar lobe (FP), F7, F3, Fz, F4, and F8 for the frontal lobe (F), C3, Cz, and C4 for the central lobe (C), T3, T4, T5, and T6 for the temporal lobe (T), P3, Pz, and P4 for the parietal lobe (P) as well as O1 and O2 for the occipital lobe (O). Furthermore, MSFME data of all electrodes were averaged to gain information about the total brain regularity (total cortex, TC). To minimize the influence of signal noise as well as the interference of possible outliers (Whitley and Ball, 2002) and due to a rather small number of subjects in each group, the median of each condition’s MSFME was used for further condition analysis. To reduce the amount of possible statistical tests regarding the number of scale factors to a relevant minimum, only MSFME values of scale factors with noteworthy differences between conditions were considered for further analysis. This procedure is based on the assumption that additional movement noise induced by means of different coordinative resp. cognitive demand takes influence on the level of EEG signal irregularity (Abhang et al., 2016). Thus, for each post-rest minute (Post1–5 and Post25) and brain area of interest (TC, FP, F, C, T, P, and O), a condition independent heterogeneity measure was implemented. This measure was defined by the calculation of the sum of MSFME differences between each condition’s median pair for each scale factor (∑_*i*_ | Δ_*i*,*j*_|) (Figure 3). The related MSFME values of the scale factor representing the maximum of heterogeneity over all scales (*max* (∑_*i*_ | Δ_*i*,*j*_|)) were used for the consecutive statistical analysis.

Concerning the interpretation of entropy in this study, it is refrained from a direct relation of high entropy to complexity. Entropy is only considered as irregularity of a time signal in its fundamental sense. Complexity is seen as balance between order and disorder resp. regularity and irregularity (Fernández et al., 2014). Absolut irregularity, i.e., highest entropy, represents pure chaos, but the amount of information needed to describe the investigated system, i.e., complexity (Prokopenko et al., 2009), is quite low (Gershenson and Fernandez, 2012). To investigate concrete complexity of a signal, applying further measures beside entropy, going beyond the scope of this study, would be obligatory.

#### Correlation of Multiscale Fuzzy Measure Entropy

To analyze an interaction effect between the heart and brain signal irregularity, Spearman correlation of time and amplitude-normalized EEG and ECG MSFME according to pre-defined scales was applied. To handle the different underlying time characteristics of the heart and brain signal, MSFME EEG and ECG values with differing scale factors were used for the correlation analysis. For each post-rest minute (Post1–5 and Post25) and brain area of interest (TC, FP, F, C, T, P, and O), the following procedure was conducted: The determination of EEG and ECG scale factor was set via a MLA condition independent heterogeneity measure (Figure 3), calculating for each scale factor the sum of MSFME differences between each MLA condition’s median pair (∑_*i*_ | Δ_*i*,*j*_|). The related MSFME values of the scale factor representing the maximum of heterogeneity over all scales (*max*⁡(∑_*i*_ | Δ_*i*,*j*_|)) were used for the following EEG and ECG correlation analysis. For the correlation, all 10-s MSFME intervals of the relevant post-rest minute were included.

### Data Analysis

According to Fisher statistics, a statistical significance level of five percent (*p* ≤ 0.05) was set. No claim to generalization of results is followed, instead results are taken to be a sufficient basis to encourage further similar studies. Calculation of effect size relied on Neyman-Pearson statistics (Neyman and Pearson, 1933) and results were presented by Cohen’s *d* (Cohen, 1988): *d* < 0.5 (small effect), *d* < 0.8 (medium effect), *d* > 0.8 (large effect). Processed data and all the other measured data were entered into the software SPSS 23 (IBM, Armonk, NY, United States) for statistical analysis. All conducted statistical tests were non-parametric due to non-standard normal distributed data and to minimize possible influence of artifacts. Based on rank scaling, the interference of possible outliers in data analysis is reduced (Whitley and Ball, 2002). Descriptive statistics were compiled for every subregion of analysis (Table 2).

**TABLE 2:** Descriptive statistics of selected variables.

		DL0	DL005	DL01	DL1
**N**		7	8	8	8
**Age**		28 (6)	28.5 (7)	27.5 (7)	27 (8)
**HR (Δ%)**		112.6 (37.4)	128.5 (33.9)	131.8 (48.4)	129.7 (56.7)
**RPE (6–20)**		15 (2)	12 (3)	12.5 (4)	15.5 (4)
**Interruptions**		10 (12)	12.5 (8)	17.5 (4)*DL0	23 (15)*DL0
**MSFME (Δ%)**					
**EEG**					
***TC***	Post1	6.2 (12)	–3.5 (10.6)	0.3 (13.1)	–5.5 (14.3)
	Post5	3.8 (14.3)	–2.3 (17.6)	0.1 (8.2)	–5.4 (8.3)
	Post25	–8.5 (11.6)	–6.5 (12)	–5.8 (22.3)	–1 (10.9)
***FP***	Post1	9.9 (48.4)	–11.9 (35.5)	–3.1 (46)	–9.4 (20.3)
	Post5	–1.7 (61.1)	–8.1 (33.1)	6.5 (31.5)	–3.1 (16.7)
	Post25	–0.3 (50.5)	–6.4 (22.9)	–2 (24.1)	–2.1 (14.5)
***F***	Post1	7.1 (14.8)	–6.2 (11.8)	3.1 (24)	–4.4 (13.6)
	Post5	–0.7 (12.8)	–3.6 (14)	5.5 (11.8)	–4.7 (15.7)
	Post25	–9.6 (14.7)*	–6.3 (7.3)	–9.4 (24.5)	–2.8 (18)
***C***	Post1	8.6 (19.4)	2.9 (41.8)	8.2 (12)	0.6 (7.7)
	Post5	4.4 (22.5)	2.9 (23.9)	–3.7 (16.4)	–7.6 (14.2)*
	Post25	–7.1 (4.5)*	–5.8 (13.1)	0.6 (19)	–1 (15.3)
***T***	Post1	–2.3 (16.8)	–8.4 (4.6)	–6.7 (10.6)*	–3.9 (14)
	Post5	–4.8 (13.5)	–1 (18.2)	–0.7 (9)	5.9 (19.7)
	Post25	–13.6 (11.8)*	–3.7 (12.4)	–7 (19.3)*	–2.1 (18.8)
***P***	Post1	8.1 (22.7)	–1.2 (8.9)	–8 (10.8)	–1.9 (5.5)
	Post5	4.5 (17.5)	1.3 (22.1)	–2.2 (7.2)	–6.6 (8.4)*
	Post25	–2.5 (3.7)	–0.7 (9.2)	–9.5 (11.7)*	–4.1 (14.5)
***O***	Post1	19.4 (28.4)*	11.1 (34.7)	3.1 (23.3)	3.2 (9.8)
	Post5	4.2 (12.1)	2.6 (20.6)	–6.1 (19.3)	–6.7 (7.5)
	Post25	13.5 (28.2)*	5.6 (35.9)*	2.6 (29.8)	–6.3 (11.8)
**ECG**	Post1	26.3 (24.7)*	2 (55.1)	31 (76.8)	12.4 (59.4)
	Post5	7.2 (33.9)	28.3 (28.8)*	24.2 (32)	11.2 (51.6)
	Post25	11.9 (14.9)	22.8 (28.1)	20.5 (23.7)	4.3 (24.8)

Columns defined by motor learning approaches, rows defined by behavioral and MSFME EEG and ECG variables. EEG MSFME divided by cerebral lobes. Median (IQR) values, Δ% defining relative change to baseline. *Signifies significant time effect to baseline. *DL0 signifies significant difference to DL0 motor learning approach.

#### Behavioral Data and Borg Scale of Perceived Exertion

To check for possible differences in age, RPE as well as number of rope skipping interruptions between all conditions, Kruskal-Wallis test including *post hoc* Bonferroni correction was used.

#### Multiscale Fuzzy Measure Entropy

The following statistical tests were conducted for each EEG brain area as well as ECG dependent MSFME values: To statistically evaluate the acute training intervention and short-term recovery effects, the Wilcoxon test comparing the baseline with each of the first five post-rest minutes was used for each training intervention. A possible remaining effect of an intervention after recovery was examined by comparing the baseline with the last post-rest minute by means of the Wilcoxon test. Regarding a direct comparison between training interventions in acute short-term, i.e., first five post-rest minutes, and at the end of recovery, i.e., last post-rest minute, the Kruskal-Wallis test with Bonferroni *post hoc* test was chosen.

#### Correlation of Multiscale Fuzzy Measure Entropy

For calculation of an interaction effect of brain and heart signal irregularity, Spearman correlation was used due to nonparametric data and to the advantage of minimizing the effect of possible outlier measurement data. Only Spearman correlation coefficients (*r*_*s*_) with *r*_*s*_ ≥ 0.3 (moderate relation) were considered for the further presentation of results and their interpretation. Correlation coefficients *r*_*s*_ ≥ 0.5 represent a strong relation (Cohen, 1992).

## Results

### Behavioral Data and Borg Scale of Perceived Exertion

Statistical analysis showed no significant differences in age, RPE and heart rate intensity of the training interventions between MLAs. Only the number of interruptions in rope skipping significantly differed between the training interventions. Post hoc analysis showed significantly more interruptions in DL1 and DL01 compared to DL0.

### Multiscale Fuzzy Measure Entropy

The statistical analysis of MSFME during the first five and the last post-rest minutes yielded neither in EEG nor in ECG any significant effects in condition comparison.

Analysis of time effects regarding the acute and short-term as well as remaining impact led to condition dependent significant effects (Table 3). The investigation of time effects in MSFME regarding the first 5 mins after rope skipping revealed condition, and in relation to EEG, brain lobe dependent effects. DL0 showed directly at the beginning of post-rest session an increase of O MSFME. In the 3rd post-rest minute, there was a reduction of MSFME in F. More statistically significant effects were found in DL005. A reduction of MSFME in TC as well as particularly in F and O were observed already during the 2nd post-rest minute. The effect in O remained till the 3rd post-rest minute accompanied with an increase of T MSFME. DL01 decreased as the only significant change its T MSFME immediately following rope skipping. The condition with the latest present changes was defined by DL1. Not until the 3rd post-rest minute, a reduction in P and an increase in T regions were determined. Later on, a reduction of C MSFME was added. Till the end of the 5th post-rest minute, C and P MSFME stayed reduced. Analysis of ECG MSFME revealed significant increases in all conditions except DL01 dependent on time of first occurrence. DL0 led immediately after rope skipping to an increase. But this increment only prolonged till the end of the 2nd post-rest minute. Following DL0, DL1 showed a brief increment in the 2nd post-rest minute. Similarly in occurrence time, the most prolonged increase of ECG MSFME, till the end of the 5th post-rest minute, was observed in DL005.

**TABLE 3:** Significant time effects of EEG and ECG multiscale fuzzy measure entropy (MSFME) changes.

		Post1			Post2			Post3			Post4			Post5			Post25		
		*z*	*p*	*d*	*z*	*p*	*d*	*z*	*p*	*d*	*z*	*p*	*d*	*z*	*p*	*d*	*z*	*p*	*d*
**EEG**																			
**DL0**	***F***							–2.197	0.028	2.981							–2.028	0.043	2.387
	***C***																–2.366	0.018	3.996
	***T***																–2.366	0.18	3.996
	***O***	2.028	0.043	2.387													2.028	0.043	2.387
**DL005**	***TC***				–1.96	0.05	1.922												
	***F***				–1.96	0.05	1.922												
	***T***							1.96	0.05	1.922									
	***O***				–2.24	0.025	2.594	–2.1	0.036	2.217									
**DL01**	***T***	–2.1	0.036	2.217													–1.96	0.05	1.922
	***P***																–2.1	0.036	2.217
**DL1**	***C***										–2.1	0.036	2.217	–1.96	0.05	1.922			
	***T***							2.1	0.036	2.217									
	***P***							–2.1	0.036	2.217				–2.38	0.017	3.115			
**ECG**																			
**DL0**		2.366	0.018	3.996	2.028	0.043	2.387												
**DL005**					2.38	0.017	3.115	2.24	0.025	2.594	2.1	0.036	2.217	2.1	0.036	2.217			
**DL01**																			
**DL1**					2.1	0.036	2.217												

*Columns defined by post-rest minutes of interest (Post1–5 and Post25). Rows defined by EEG and ECG MSFME, each divided in conditions, EEG MSFME additionally divided in cerebral lobes with significant changes. Values of statistical tests.*

The analysis of a remaining effect after total recovery compared to baseline (Table 3) led alike to significant changes with condition differences, at least regarding EEG data. DL0 kept at the end of recovery the significant changes of short-term recuperation in F and O stable, whereas a reduction in C and T was added. DL05 reduced its short-term recovery effects to only a remaining decrease in O MSFME. DL01 added to its decrease in T, also a reduction in P. In contrast to all other conditions, no remaining effects were found in DL1 indicating a reset of MLA related rope skipping effects. Regarding ECG, at the end of recovery there were no sustained significant effects.

### Correlation of Multiscale Fuzzy Measure Entropy

The correlation analysis of brain area dependent EEG and ECG MSFME revealed condition specific differences (Table 4). Fewest relations were found in DL0 with general lowest absolute relation level compared to other conditions. The effects were only of negative correlation with a medium relation, firstly present in C of the 2nd post-rest minute and later in P and TC. DL005 led also to mainly negative relations, but with partially strong effects and different involved brain regions. Particularly, FP yielded from the 1st till the end of the 4th post-minute session constantly a negative relation to ECG MSFME. F and P regions showed negative relation in the 4th post-minute. The only positive correlation was in the 5th post-rest minute with a strong relation of O and ECG. Even more brain lobes were moderately related in DL1 with mainly positive relations at least till the end of the 4th post-rest minute. In the 2nd post-rest minute, C, P, and O as well as in the 4th post-rest minute, FP and T led to positive relations to ECG MSFME. Only in the 5th post-rest minute, negative relations were determined in TC as well as C and P. The most and highest correlation effects were ascribed to the DL01 condition. In all brain regions except F, mainly positive relations of EEG and ECG MSFME were found. The only negative correlation was in T in the 1st post-rest minute. Moderate positive relations emerged in TC, C, and P from the 3rd post-rest minute on and in O in the 5th post-rest minute. A stable and even mostly strong relation of EEG and ECG MSFME over the whole recovery time, including the ultimate post-rest minute, was observed in FP. No remaining, at least moderate relations of EEG and ECG MSFME were assessed in the other conditions.

**TABLE 4:** Correlation results of EEG and ECG MSFME.

		Post1	Post2	Post3	Post4	Post5	Post25
**DL0**	***TC***					–0.337	
	***C***		–0.314			–0.34	
	***P***				–0.409		
**DL005**	***FP***	–0.459	–0.39	**-0.543**	**-0.541**		
	***F***				–0.436	–0.483	
	***P***				–0.38		
	***O***					**0.567**	
**DL01**	***TC***			0.331	0.322		
	***FP***	0.377	**0.629**	**0.522**	**0.787**	**0.633**	0.329
	***C***			0.406	0.416	0.386	
	***T***	–0.318					
	***P***			0.421		0.321	
	***O***					0.408	
**DL1**	***TC***					–0.371	
	***FP***				0.348		
	***C***		0.446			–0.303	
	***T***				0.426	**0.518**	
	***P***		0.32			–0.504	
	***O***		0.411				

*Columns defined by post-rest minutes of interest (Post1–5 and Post25). Rows defined by the conditions, subdivided in cerebral lobes (TC, FP, F, C, T, P, and O) with at least moderate effects (r_s_ ≥ 0.3). Spearman correlation coefficient r_s_ values, strong effects (r_s_ ≥ 0.5) in bold.*

## Discussion

This study aimed to investigate and compare the influence of a cyclic and coordination demanding physical activity (PA) of medium physical load with different underlying motor learning approaches (MLA) on brain and heart activity by means of a nonlinear analysis via multiscale fuzzy measure entropy (MSFME). Furthermore, brain and heart interactions were examined via the correlation of EEG and ECG MSFME. An increased general EEG and reduced ECG MSFME in all motor learning conditions immediately after rope skipping at rest was hypothesized. In recovery, MSFME of EEG and ECG may change slowly towards pre-rest value. Referring to an effect of the MLAs, we assumed higher general MSFME in DL005, DL01, and DL1 due to additional cognitive workload compared to DL0. Furthermore, differences in the interaction of EEG and ECG MSFME via correlation analysis between MLAs were hypothesized immediately following rope skipping and in subsequent recovery. For interpretation of results, it was resorted to the possible direct link between power spectrum and entropy (Lin et al., 2017).

The analysis of behavioral data as well as perceived exertion did not significantly differ between MLAs indicating a similar physical load. Nevertheless, the number of interruptions, was significantly different, particularly in DL01 and DL1 with more interruptions compared to DL0. This is in line with a much higher coordinative demand in DL approaches according to the additional movement tasks. Regarding the descriptive data, a linear relationship between the number of movement interruptions and the frequency of task instructions could be assumed. However, since the number of interruptions was small compared to the number of correctly performed movements and no significant difference between behavioral as well as perceived exertion between conditions was found, a dominant influence of movement variety on the EEG and ECG effects could be speculated.

### Motor Learning Approach Dependent Effects in Electroencephalography and Electrocardiography Signal Irregularity

Recapitulating, a lower entropy indicates a more predictive signal with a high level of regularity, whereas higher entropy suggests a less predictive signal with less regularity (Akaike, 1985; Lin et al., 2017). Irregularity analysis immediately after rope skipping revealed MLA dependent different time effects in EEG and ECG MSFME. The hypothesized general increase in EEG and decrease in ECG MSFME independent of MLAs was not found. Furthermore, the hypothesized general changes in recovery toward pre-rest level could not be identified. Regarding the short-term recovery, i.e., first 5 mins of recovery, MLAs showed in both biosignals referring to the pace of occurrence differing statistically significant effects. Besides, EEG MSFME varied in the alteration, i.e., present positive as well as negative changes, and in the brain lobes. Additionally, even after the fully analyzed recovery time of 25 min, there were still remaining MLA dependent significant EEG MSFME effects. In contrast, ECG MSFME led to no remaining significant effects independent of MLA indicating a complete metabolism related recovery of the rope skipping induced changes after total recovery time. All these general statements point to a specific impact of additional movement noise on brain and heart signal irregularity. Based on varying effects dependent on brain lobes and pace of effect occurrence, a more detailed interpretation of results is presented in the following.

Considering EEG MSFME, DL0 increased as the only MLA immediately after rope skipping, i.e., 1st post-rest minute, its EEG signal irregularity, in the occipital lobe (O). According to no additional movement noise in DL0 during rope skipping, subjects could perhaps have focused more on visual instead of on other types of sensory processing. DL01 in contrast decreased its signal irregularity in the temporal lobe (T) possibly indicating a reduction of auditive or emotional processing (Kolb and Whishaw, 2009). Movement tasks during rope skipping were verbally instructed, thus a higher demand of auditive sensory processing could have led to a subsequent fatigue of this functionality or a reduction of focusing to this sensory area. Whether a decreasing entropy also implies a shift in frequency composition to lower frequencies has to be examined in future research. In this context, provoked lower frequencies, e.g., theta, could lead to a wider integration of different brain regions (Caplan et al., 2003; Backus et al., 2016) which could be interpreted as a more holistic communication over various brain lobes. DL005 and DL1 resulted in a delayed change of their EEG signal irregularity, not before the 2nd and 3rd post-rest minutes. MSFME was first reduced in the total cortex (TC), the frontal lobe (F) as well as O in DL005, and even later in the parietal (P) and central lobe (C) in DL1. DL005 seems to reduce its signal irregularity in TC, particularly in F as well as O. Considering the specific brain lobes, a reduction in processes of movement selection as well as visuomotor and object-recognition functions could be assumed (Kolb and Whishaw, 2009). Regarding DL1, it could be hypothesized that only with prolonged recovery a reduction of processing in somatosensory and motor areas (Nolte, 2009) emerged. Furthermore, both MLAs revealed additionally an increase of signal irregularity in T in the 3rd post-rest minute perhaps indicating a provoked organization of sensory input (Kolb and Whishaw, 2009).

Regarding the analysis of a remaining effect at the end of the measured recovery phase (after 25 min), except DL1 all MLAs led to still significant changes compared to pre-exercise baseline. DL0 yielded the most changes in brain regions with a reduction in F, C, and T as well as an increase in O signal irregularity. In addition to hypothesizing decreased processing of motor movements and auditive or emotional information, still provoked visual processing could be assumed. Fewer changes were found in DL01 with a decrease in T and P as well as in DL005 with only an increase in O signal regularity. These changes in DL01 could suppose a reduction in the organization of sensory input as well as somatosensory processing. Total recovery time in DL005 could have led to a reduced visual processing function.

The results of ECG MSFME showed also differences between MLAs till first significant changes were determined. As the only MLA, DL0 revealed immediately after rope skipping an increase in ECG signal irregularity. Subsequently, i.e., in the 2nd post-rest minute, additionally DL1 and DL005 raised their MSFME. In respect of the following recovery, only DL005 kept a significant increase till the end of the short-term recovery, i.e., 5th post-rest minute. In contrast, DL01 provoked no significant change of its ECG MSFME during the total recovery time at all. Thus, all MLAs except DL01 seem to provoke the heart signal irregularity with different paces. Whether the specific frequency of changes in movement tasks in DL01 were responsible for its stable signal irregularity, needs further research.

### Motor Learning Approach Dependent Effects in the Correlation of Electroencephalography and Electrocardiography Signal Irregularity

The investigation of the Spearman-correlation between the brain and heart MSFME revealed different effects in MLAs dependent on the brain region and the time till first significant changes could be identified. This refers to the acute impact immediately after rope skipping, to the subsequent short-term recovery and the control of any remaining effects after the total recovery phase. Hence, the hypothesis regarding a different impact of MLAs on the MSFME EEG and ECG correlation received corroboration. For a first rough impression, the focus was given only to the most prominent correlation effects for interpretation. Therefore, only results with at least moderate correlation effects were considered independent of their sign because both indicate a closer relationship between the two entropy signals.

DL0 as the MLA with no additional movement noise revealed the fewest, latest, smallest, and solely negative correlation effects in comparison of MLAs. The signal irregularity of C correlated moderately negatively in the 2nd and 5th post-rest minute, P in the 4th and TC not until the 5th post-rest minute with the heart. DL005, MLA with the lowest grade of additional coordinative demand, revealed noteworthy effects in more brain lobes, in the frontopolar lobe (FP) particularly from beginning of recovery on, and even with partly strong effects. Except a strong positive effect in O in the 5th post-rest minute, all other noteworthy correlations of FP, F, and P with the heart were negative. The most, fastest, and highest correlation effects over all brain areas except F were found in DL01, the MLA with medium additional coordinative load, with the particular characteristic of mainly positive correlations. Especially, correlations of FP mainly representing strong relations to the heart in all measurement times were prominent. FP of DL01 was additionally the only remaining effect induced by a MLA after the total recovery time. The coordinatively most demanding MLA, DL1, yielded in the same brain regions as in DL01 fewer and slightly later as well as smaller correlations. Furthermore, beside several positive correlations, negative correlation effects increased compared to DL01. Summarizing, the most correlation effects were found in FP, C, and P. Thus, these lobes and their underlying functions seem to be prominent in the interaction with the heart, at least related to the non-linear analysis used and as a consequence of rope skipping with additional movement noise. Regarding this, a particular involvement of the central autonomic network (CAN) could be suggested. The CAN represents a complex of brain structures and connections controlling the autonomic regulation in multiple physiological conditions (Silvani et al., 2016; Valenza et al., 2019; Candia-Rivera et al., 2021). Particular parts of the CAN are the FP, middle, and posterior cingulate cortex, which’s brain activity were positively associated with the instantaneous heart rate at rest (Valenza et al., 2019). These brain structures could correspond in a way to the most prominent structures in this study, if hypothesizing the measurement of C and P electrical activity contains signal content of the deeper lying middle and posterior cingulate cortices. FP is among others assumed to be responsible for the top-down control of executive functions like selective attention, self-control and -regulation (Robertson and Marino, 2016). In this context, the correlations of these brain regions with the heart could indicate a provoked interaction of the top-down control of executive functions and the heart signal irregularity dependent on the additional movement noise level given. Because statistical correlation contains no information about the cause-effect relationship, whether the FP area is more sensitive to the heart signals or the other way round needs to be studied in future too. Nevertheless, it could be hypothesized that an activation of FP could possibly indicate an indirect training of the executive functions by means of physical activity which would be beneficial besides sports in other life areas. Considering this study, including additional movement noise could probably lead to an even higher and faster demand of structures that support certain executive functions, but only to a specific limit of additional movement noise.

In the search for possible underlying neurophysiological structures that could be responsible for some of the observed phenomena, the nucleus locus coeruleus (LC) could be a primary candidate. The LC is the near exclusive source of Noradrenalin in cortical areas and not only projects to the forebrain (Chandler, 2016) and motor areas (Ludwig, 2020) but also augments inhibitory transmission to parasympathetic cardiac vagal neurons (Wang et al., 2014). In the waking state, two modes of LC function are distinguished (Aston-Jones and Cohen, 2005). In the phasic mode, short bursts (<300 ms) of higher frequency activity (10–15 Hz) regulate the encoding of salient stimuli (Vazey et al., 2018). The tonic mode is characterized by an irregular but continuous baseline activity (1–6 Hz) and associated with exploratory and highly distractable behavior as well as with reduced task utility or stress (Aston-Jones and Cohen, 2005). Interestingly, phasic activity is absent with a low and high tonic LC baseline (Janitzky, 2020). Only medium tonic activity, as e.g., in exercising settings (Dietrich and Audiffren, 2011; Audiffren, 2016), is associated with phasic activity that is essential for optimal neural network function (Janitzky, 2020). According to this study, the conducted rope skipping frequency of approximately 2 Hz could interfere via the resonance effect with the present averaged pulse frequency around 2.5 Hz. This could result in potential changes of the autonomic nervous system (ANS) and subsequently influence the ANS related brain processes (Ako et al., 2003; Tang et al., 2009; Garcia et al., 2011; Kuo et al., 2016; Triggiani et al., 2016). All together would provide a plausible model for an inverted U-shape relationship (Aston-Jones and Cohen, 2005) between the level of additional movement noise during load and the heart-brain relaxation behavior. Interestingly, the relationship between noise level and learning rate also shows an inverted U-shape (Schöllhorn, 2005; Schöllhorn et al., 2006, 2009). Further studies will have to show to what extent the same causes underlie both. The possible scope of the consequences would be worth the effort.

In addition, based on the vertical deflection of the total body during rope skipping, other neuronal and mechanical stimulations with similar frequencies become candidates for interactions. Besides direct rhythmic neuronal activations of somatosensory and motor areas by cyclic activation of the leg muscles, the modification of primarily muscular “microvibrations” (Rohracher, 1962), the mechanical stimulation by means of the pulse wave traveling through the blood vessels of the brain, as well as the whole body-tissue stimulation by the cyclic impacts have to be distinguished. In this context, the physiological effects are highly dependent on the type of underlying stimulation. Whole-body vibrators usually stimulate neuronal activation of cortex areas via tonic muscle activation and modification of “microvibrations” with varying stimulation frequencies of 1–25 Hz at a vertical amplitude of up to 2.5 cm (Taiar et al., 2018) and an only slightly increased heart rate (Furness et al., 2014). The frequency during rope skipping was approximately 2 Hz, the averaged heart rate around 150 bpm and the vertical amplitude at appr. 15 cm, leading to significantly increased accelerations of the head and the brain tissue within it including all blood vessels. Consequently, due to the specific skipping characteristics and the level of cardiovascular intensity as well as the new information inflow by means of additional movement noise, a parallel impact of rope skipping on LC activity and heart rate could be assumed. Future research must show to what extent phase shifts of the signals have an effect. Similar frequencies around 2 Hz in disco beat, folk and shamanic dances as well as in pendulum swinging in preparation for hypnosis suggest a more general significance of this frequency. To what extent this phenomenon can be harnessed for prophylaxis in Parkinson’s and Alzheimer’s disease due to their dependence on LC will be revealed by future research (Janitzky, 2020).

As the prior rope skipping study (John and Schöllhorn, 2018) suggested, high levels of additional movement noise could lead to emotional and cognitive overload. The comparison of the underlying MLAs investigated supports this hypothesis. But, instead of the highest level of additional movement noise, the far lower additional movement noise defined by DL01 seems to produce in the subsequent recovery the highest interaction level between the FP area and the heart signal irregularity. C, i.e., the primary motor cortex, is associated with the execution of motor movements and P to the sensory perception of the body, somatosensory processing and motor learning (Penfield and Boldrey, 1937; Nolte, 2009). Compared to FP, a in a broader sense similar, but slighter trend of C as well as P interaction with the heart could be assumed dependent on additional movement noise.

**FIGURE 4:**
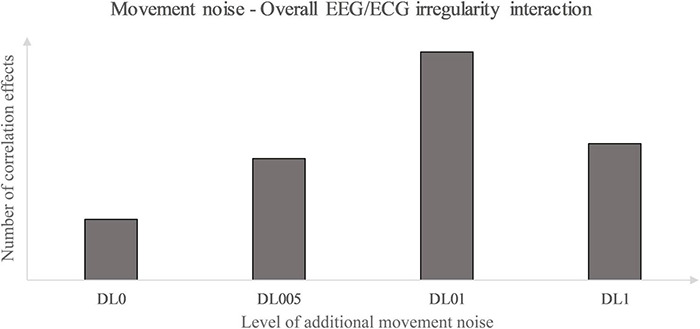
Movement noise – electroencephalography (EEG)/ electrocardiography (ECG) irregularity interaction.

**FIGURE 5:**
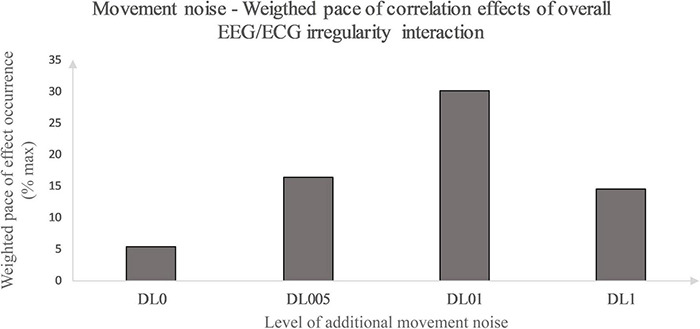
Movement noise – Weighted pace of correlation effect occurrence relative to maximal weighted pace. Weighted pace calculated via weighting at least moderate correlation effects according to their occurrence in time. Effect occurrence was weighted as followed: 1st post-rest minute – value 1, prior weighted pace value divided by 2 defining pace value of subsequent post-rest minute. Weighted pace values of each post-rest minute of interest and brain lobe were summarized for all conditions and divided by the maximal weighted pace possible (% max).

Primarily, this study used the interaction analysis to identify concatenations between the brain, differentiated by brain lobes, and the heart dependent on physical and coordinative load. Interactions of the (neuro-)physiological systems are seen to be fundamental for sustaining homeostasis in a continuously changing environment (Chang et al., 2016). Elucidating that positive as well as inverse correlation effects represent a type of communication between the two biosignals, both correlation types are seen as some sort of concatenation between the heart and the brain activity. Considering this, the correlation type could be disregarded in a first approach and an overall measure of concatenation could be implemented. Thus, the amount of each underlying MLA’s correlation effects between the EEG and ECG MSFME, independent of the recovery time, could be modeled in general as an inverted U-shape with the level of coordinative demand on the X-axis and the number of at least moderate correlations on the Y-axis (Figure 4). Previous EEG research based on linear analysis already supposed the existence of an inverted U-shape regarding the cognitive functioning, i.e., working memory, based on exercise intensity (Kamijo et al., 2004) and regarding the spectral power based on exercise duration related to emerging fatigue (Gronwald and Hottenrott, 2013). In this case, it would indicate that an optimum trend exists that neither no nor the highest amount of additional movement noise, but a specific amount in between lead to a highest concatenation between brain and heart signal irregularity. Referring to the time till first noteworthy correlation effects appear, a similar inverted U-shape, but with the Y-axis defined as the weighted pace of correlation occurrence could be suggested (Figure 5).

### Limitations

This study took a more holistic approach to investigate the interaction of the heart and brain as a function of physical and coordinative stress. Due to the novelty, only a rough impression of the complexity between the two biosignals could be aimed at. Many more questions arose that need to be investigated in the future. Due to the chosen design and statistics, no claim to general validity is made. Each of the following limitations holds the potential to explore a different criterion and expand our understanding of human complexity.

Considering the determination of the underlying DL MLAs, particularly the frequency of new tasks and their absolute number, there is an enormous span between DL01 with 18 tasks and DL1 with 180 tasks in total. In respect to the study results and the derived inverted U-shape representing the general brain heart interaction dependent on the level of additional movement noise, another underlying MLA condition with a task frequency somewhere between DL01 and DL1 would provide more detailed insight to underpin this suggestion. In this context, comparing the influence of an intervention with various movement tasks only once executed with an intervention of a limited number of differing, but periodically switching movement tasks would be of interest, too. More specifically, the contextual interference approach (Magill and Hall, 1990) as an underlying MLA would lead to, e.g., three skipping exercises between those the subjects would have to switch either in blocked, serial, or random order in the applied time schedule every 10 or 20 s. Accordingly, the impact of ever new compared to differing, but repeated information could be investigated. In addition, whether the supposed inverted U-shape of brain heart interaction in general would also fit to the concatenation level between each single brain lobe and the heart needs to be clarified in the future. Further on, the influence of rope skipping interruptions on EEG and ECG compared to the influence of exercise number has to be partialized out in future research. Furthermore, with respect to the method of analysis, the setting of data interval length and entropy calculation parameters could have influenced the analysis output. Based on the alternative research character, the setting of parameters was premised on the recommendations of previous studies. Regarding the data interval length, the different underlying time scales of the brain and heart signal aggravate their comparison. Therefore, an interval length was chosen that was assumed to be adequate to include enough time characteristics especially of the rather slow heart signal. Future research should address the investigation of the impact of varying parameter settings and data interval lengths. Regarding the MSFME calculation, the determination of scales of interest via the heterogeneity measure used represents only a first possibility to reduce the huge data amount to a relevant minimum. Considering this, it is fundamental to declare that this procedure relies on the prerequisite that physical activity leads according to varying additional movement noise to different outputs in signal irregularity. Nevertheless, enough incentives to this analysis application were given by the EEG spectral power results of the prior rope skipping study (John and Schöllhorn, 2018) and a study indicating similar EEG entropy as their spectral power results (Lin et al., 2017). However, this direct relation between EEG spectral power and entropy still has to be handled with caution and it strives for further research. Further on, the applied interpretation type of entropy, i.e., solely as irregularity of a time signal, represents only one way of entropy explanation. Despite and especially because of all the limitations, all the findings together promote a more holistic analysis of the complex interactions in our bodies during and due to physical activity.

## Data Availability Statement

The raw data supporting the conclusions of this article will be made available by the authors, without undue reservation.

## Ethics Statement

The studies involving human participants were reviewed and approved by Local Institutional Ethics Committee Institute of Sport Science Johannes Gutenberg-Universität Mainz. The patients/participants provided their written informed consent to participate in this study.

## Author Contributions

All authors listed have made a substantial, direct, and intellectual contribution to the work, and approved it for publication.

## Conflict of Interest

The authors declare that the research was conducted in the absence of any commercial or financial relationships that could be construed as a potential conflict of interest.

## Publisher’s Note

All claims expressed in this article are solely those of the authors and do not necessarily represent those of their affiliated organizations, or those of the publisher, the editors and the reviewers. Any product that may be evaluated in this article, or claim that may be made by its manufacturer, is not guaranteed or endorsed by the publisher.
